# Security at the Edge for Resource-Limited IoT Devices

**DOI:** 10.3390/s24020590

**Published:** 2024-01-17

**Authors:** Daniele Canavese, Luca Mannella, Leonardo Regano, Cataldo Basile

**Affiliations:** 1 IRIT, CNRS, 118 Route de Narbonne, CEDEX 9, F-31062 Toulouse, France; 2 Dipartimento di Automatica e Informatica, Politecnico di Torino, Corso Duca degli Abruzzi 24, 10129 Turin, Italy; 3 Dipartimento di Ingegneria Elettrica ed Elettronica, Università degli Studi di Cagliari, Piazza d’Armi, 09123 Cagliari, Italy

**Keywords:** authentication, cybersecurity, edge computing, Internet of Things (IoT), intrusion prevention system (IPS), machine learning, gateways, oblivious authentication, proxy, virtual private network (VPN)

## Abstract

The Internet of Things (IoT) is rapidly growing, with an estimated 14.4 billion active endpoints in 2022 and a forecast of approximately 30 billion connected devices by 2027. This proliferation of IoT devices has come with significant security challenges, including intrinsic security vulnerabilities, limited computing power, and the absence of timely security updates. Attacks leveraging such shortcomings could lead to severe consequences, including data breaches and potential disruptions to critical infrastructures. In response to these challenges, this research paper presents the IoT Proxy, a modular component designed to create a more resilient and secure IoT environment, especially in resource-limited scenarios. The core idea behind the IoT Proxy is to externalize security-related aspects of IoT devices by channeling their traffic through a secure network gateway equipped with different Virtual Network Security Functions (VNSFs). Our solution includes a Virtual Private Network (VPN) terminator and an Intrusion Prevention System (IPS) that uses a machine learning-based technique called oblivious authentication to identify connected devices. The IoT Proxy’s modular, scalable, and externalized security approach creates a more resilient and secure IoT environment, especially for resource-limited IoT devices. The promising experimental results from laboratory testing demonstrate the suitability of IoT Proxy to secure real-world IoT ecosystems.

## 1. Introduction

The Internet of Things (IoT) domain is continuing to grow. In 2022, IoT Analytics [1] estimated 14.4 billion active endpoints despite the recent chip shortage. Moreover, they forecast approximately 30 billion connected devices in 2027. This massive number of machines has facilitated seamless data sharing and access to services worldwide, fundamentally altering how we interact with technology. Indeed, those devices are becoming very popular even in our homes. Around 40% of houses worldwide have an IoT device. Considering only North America, this number grows to approximately 70% [2].

However, the rapid growth of IoT devices has come with significant security challenges. These interconnected devices, equipped with myriad sensors that capture data from their surroundings, generate vast amounts of information. Unfortunately, many IoT devices suffer from intrinsic security vulnerabilities due to limited computing power, low-cost design, and the absence of timely security updates [3]. In addition, even software developers can play a significant role in introducing security vulnerabilities in IoT ecosystems since they may not be fully aware of the potential threats that could affect their IoT project [4]. Indeed, many hobbyist open-source IoT projects contain potential issues [5].

These shortcomings create opportunities for malicious actors to exploit vulnerabilities, e.g., weak or hard-coded passwords, inadequate encryption mechanisms, or insecure communication protocols. Consequently, compromised IoT devices can serve as entry points for unauthorized access, leading to severe consequences, including data breaches and potential disruptions to critical infrastructures. The urgent need for robust security measures in the IoT ecosystem is exemplified by the rise of cyber attacks that target IoT devices. In 2016, the discovery of the infamous Mirai malware [6] demonstrated the potential of IoT devices being turned into remotely controlled bots, facilitating large-scale Distributed Denial of Service (DDoS) attacks. The consequences of such attacks could be devastating and even endanger human lives, impacting essential services and healthcare systems.

The challenges associated with securing IoT devices are further complicated by factors such as the diversity of devices and manufacturers, making it challenging to establish uniform security standards and practices. Manufacturers often prioritize time-to-market and cost efficiency over comprehensive security measures, leaving IoT devices susceptible to exploitation. The result is a gap between the rapid expansion of IoT and the development of robust security measures to safeguard these devices.

In response to these challenges, this research paper presents the *IoT Proxy*, a modular component designed to enhance (resource-limited) IoT devices’ security. As part of the FISHY project^1^ (funded by the European Union’s Horizon 2020 research and innovation program^2^), the IoT Proxy is a critical component of a coordinated cyber-resilient platform focused on establishing trusted supply chains. The core idea behind the IoT Proxy is to externalize security-related aspects of IoT devices by channeling their traffic through a secure network gateway equipped with different Virtual Network Security Functions (VNSFs). This gateway, operating on a more powerful machine (w.r.t. resource-limited IoT devices), enforces a cascade of security controls to bolster individual device security and prevent threats from spreading throughout the network.

Due to its modular design, the IoT Proxy could include many VNSFs. In the context of this paper, we will focus on the main security functionalities: a Virtual Private Network (VPN) terminator (based on strongSwan^3^) and an Intrusion Prevention System (IPS) based on machine learning techniques. A noteworthy feature of the IPS proposed in this paper is the capability of executing the *oblivious authentication* of the connected IoT devices. Indeed, the IoT Proxy can identify which devices are trying to use its services through their network traffic profiles. In this way, authentication can be achieved without additional computational overhead on edge devices, making its adoption compatible with the typical resource constraints of IoT ecosystems.

Finally, IoT Proxy can be wholly virtualized, allowing complete scalability and adapting hardware resources according to specific workloads.

In summary, the main contributions of this paper are:

- introducing a modular platform for protecting resource-limited IoT devices;
- introducing a transparent security approach for IoT devices (and end-system users);
- introducing an authentication approach based on machine learning techniques (i.e., the oblivious authentication) with no additional computational overhead for the IoT devices.

The rest of this paper is structured as follows. In Section 2, the paper presents the state of the art in IoT security. Section 3 describes the main components of the IoT Proxy, while Section 4 highlights the promising results obtained from laboratory testing. Then, in Section 5, we conclude the paper by providing some insights about future work.

## 2. Related Work

The advent of the Internet of Things (IoT) has ushered in a remarkable era of innovation, with applications spanning from smart homes to industrial automation. However, this proliferation of interconnected devices has brought to the forefront a pressing concern: how to manage security, especially for legacy and resource-limited devices. Indeed, IoT devices, often constrained by limited computing power and memory, have become enticing targets for malicious actors seeking to compromise networks or gain unauthorized access. Safeguarding these devices and the networks they inhabit is of paramount importance.

One avenue explored in related research is using Network Function Virtualization (NFV) techniques [7] to bolster IoT security. Aman et al. [8] proposed a security framework that leverages NFV to establish trust in IoT systems and combat malware propagation. By categorizing IoT devices, using remote attestation [9], in three different categories (trusted, vulnerable, or compromised), they effectively employ NFV to create distinct networks for each category. Their results are promising, showcasing a 66% reduction in compromised devices within a mere 10 s time frame. Another interesting solution is the one proposed by Zolotukhin et al. [10]. They introduced an IoT defense system founded on Software-Defined Networking (SDN) [11] and NFV [12], incorporating machine learning to assess and respond to attack risks optimally. SDN’s separation of control and data planes facilitates centralized network control and traffic manipulation. Cloud-based servers emulate essential infrastructure components, such as routers and gateways, while virtual Network Security Functions (vNSFs), including firewalls and intrusion detection systems, fortify security. Moreover, analyzing another solution based on NFV and machine learning techniques, Guizani et al. [13] proposed an architecture that is empowered by NFV, featuring a learning model based on a Long Short-Term Memory (a specific type of Recurrent Neural Network) [14]. This model is capable of promptly predicting malware spread in heterogeneous IoT networks. Their solution scales effectively with network size growth, thanks to the integration of machine learning with a virtualized patching system. The ultimate objective of this study is the development of a versatile Intrusion Detection System (IDS) capable of countering diverse malware threats.

Talking about IDS and Intrusion Prevention Systems (IPSs), it is possible to find various solutions and methodologies explicitly tailored to enhance the security of IoT environments in the literature. S. M. Kasongo [15] introduced an IDS tailored to Industrial IoT (IIoT) applications [16], employing a Genetic Algorithm (GA) [17] for feature selection combined with a random forest model. Their approach achieves high accuracy, surpassing 85% across multiple classifiers. Kumar et al. [18] presented a distributed IDS, harnessing fog computing [19] to identify and mitigate Distributed Denial of Service (DDoS) [20] attacks in blockchain-enabled [21] IoT networks. Their system comprises three critical engines, facilitating effective attack detection and classification. Basati et al. [22] proposed a Network-based Intrusion Detection System (NIDS) built upon deep neural networks specifically designed for IoT networks. The Parallel Deep Auto-Encoder (PDAE) model enhances accuracy while significantly reducing computational requirements.

In addition to traditional approaches, some researchers have explored modern methodologies such as Blockchain [21] and edge computing [23] to fortify IoT security. Sharma et al. [24] introduced a blockchain-based solution for securing IoT devices, emphasizing data integrity and peer-to-peer authentication. Experimental results reveal improved security compared to conventional hash algorithms [25]. Jiang et al. [26] embraced edge computing, deploying a lightweight machine-learning algorithm for intrusion detection. Their edge intelligent gateway ensures real-time threat detection with minimal network performance impact.

Considering Virtual Private Networks (VPNs), this technology has emerged as a popular choice for securing IoT traffic, and several studies have delved into its application in this context. Raj et al. [27] developed a private VPN using a Raspberry Pi [28] as a gateway, enhancing security and scalability for home smart IoT networks. Their results demonstrate performance on par with commercial VPN solutions. Fan et al. [29] designed an IoT gateway fortified with comprehensive security mechanisms, including an IPSec [30] VPN for Transport Layer Security (TLS) [31]. Their modular framework enhances security and stability, accommodating various network access modes. Alharbi et al. [32] presented the FOCUS system, which combines fog computing and VPN technology for challenge-response authentication, effectively filtering out threats and ensuring low response latency. To conclude, Zedak et al. [33] proposed a secure architecture for remote data acquisition in IoT sensors, incorporating VPN technology to ensure secure end-to-end communication among sensors.

Our present work aspires to build upon this body of research. By adopting a modular approach and emphasizing portability through lightweight virtualization and Docker deployment [34], we aim to offer an integrated solution that consolidates multiple security controls rather than solely relying on a single technology. Our objective is to enhance the protection of IoT device networks comprehensively.

Furthermore, we improve the preliminary work by Corno and Mannella [35], which provides network protection by introducing a plugin-based modular approach for IoT gateways. Their solution, to shield the IoT devices, requires the developers of such plugins to provide a set of network requirements based on the Manufacturer User Description (MUD) standard [36]. Instead, the approach presented in this paper is completely transparent, since the IoT devices could be seamlessly protected by the IoT Proxy without the need for writing a specific network configuration.

Indeed, the security solutions (e.g., IDS, VPN) employed by our approach have already been successfully applied in IoT scenarios.

Nevertheless, to the best of our knowledge, our approach is the first one to provide a comprehensive security solution specifically tailored for IoT scenarios, for example, employing the oblivious authentication scheme to address the limited computational resources of IoT devices. The modularity of our solution ensures both adaptability to diverse IoT scenarios and its compatibility with new security solutions.

## 3. Proposed Approach

The Internet of Things (IoT) has ushered in a new era of connectivity, enabling machine-to-machine communication and offering many applications. However, the proliferation of IoT devices has raised significant security and privacy concerns. Many IoT devices are constrained by limited computational power and memory, making them attractive targets for malicious actors. The IoT Proxy project was conceived in response to these challenges.

IoT Proxy serves as a modular, secure gateway solution designed to improve the security posture of IoT device networks. Its core mission is to outsource security management tasks from the devices, acting as an intermediary between external networks and them. To ensure portability and ease of integration with other platforms (e.g., the FISHY platform in the context of the homonymous European project), the IoT Proxy harnesses the power of containerization (specifically Docker containers [34]). Each component and virtual Network Security Function (vNSF) is deployed on a dedicated Docker container. In this section, the paper explains the main components of the IoT proxy and their interactions.

The heart of the IoT Proxy is the *coordinator* module, which acts as the primary access point for IoT devices. This coordinator operates within a virtualized host environment, running a Linux-based operating system. It serves as the central command hub for IoT Proxy, responsible for interpreting configuration commands and orchestrating the various security controls. The coordinator container includes essential components such as iptables and a Python interpreter. These components are vital for configuring and managing the IoT Proxy environment, ensuring a smooth and consistent operation.

The coordinator is surrounded by a *set of vNSFs*, each providing specific security controls. These vNSFs can be dynamically activated or deactivated as needed, giving IoT Proxy its modularity and adaptability. This architectural flexibility allows custom security configurations tailored to the specific needs of IoT device networks. Adding or removing vNSFs as necessary allows IoT Proxy to adapt to evolving security requirements. This extensibility is achieved by updating a data structure within the coordinator, which maps vNSF names to their corresponding Docker images.

To facilitate communication between the coordinator and vNSFs, a *named pipe* serves as a communication channel that enables the execution of configuration commands and serves as a coordination conduit between the host system and the coordinator container, allowing a seamless exchange of instructions and configurations and ensuring that IoT Proxy operates effectively. IoT Proxy’s modular architecture makes it highly scalable and extensible.

**Figure 1:**
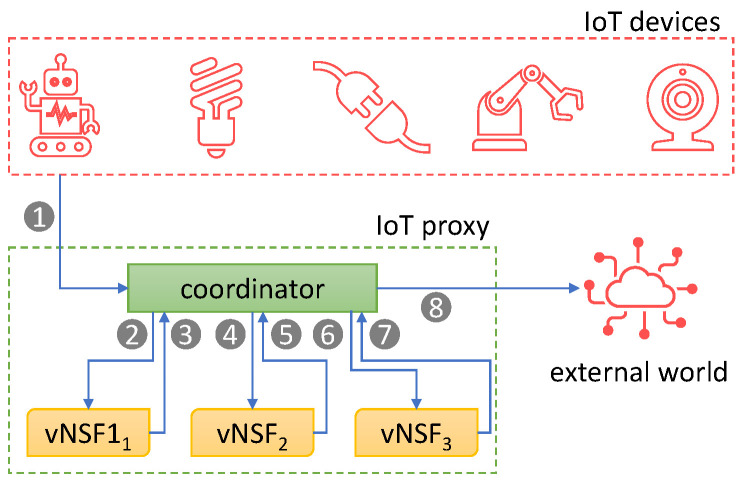
IoT Proxy high-level workflow.

Figure 1 depicts the workflow of a packet starting from an IoT device towards an external network (e.g., the Internet). The device sends the packets to the coordinator component (step 1), which acts as the proper gateway of our framework. The coordinator internally redirects all the packets to the vNSFs in order for any data analysis or traffic manipulation purposes (from step 2 to step 7). Finally, the IoT proxy emits the data to the external world (step 8). The responses to such packets will perform the same path but in a backward manner.

The versatile architecture of the IoT Proxy accommodates various security modules, making it a robust and adaptable solution for securing IoT device networks. In the current implementation, two primary vNSFs are supported: an IPsec-based VPN terminator (based on *strongSwan*^4^) and a custom Intrusion Prevention System (IPS).

### 3.1. VPN vNSF

The primary objective of the VPN vNSF is to provide encryption, authentication, and secure communication capabilities to IoT devices and networks. This module is based on *strongSwan* an open-source IPsec VPN solution. By operating at the IP layer, this module ensures IP packets’ confidentiality, integrity, and authentication, ensuring that data remain confidential and tamper-proof during transit.

IoT Proxy can seamlessly configure strongSwan instances using specific commands, making it straightforward to establish secure VPN connections for every IoT device managed by the IoT Proxy. strongSwan’s versatility is underscored by its support for various VPN types and robust encryption capabilities. It offers a spectrum of authentication methods, including Public Key Infrastructure (PKI) [37] and pre-shared keys, ensuring that IoT devices can establish secure connections. The IKEv2 [38] protocol facilitates secure key exchange, further enhancing the overall security of IoT communications.

### 3.2. IDS/IPS vNSF

Intrusion Detection Systems (IDSs) and Intrusion Prevention Systems (IPSs) frequently serve as the first line of defense to safeguard computer networks, helping to identify and respond to unauthorized accesses and malicious activities. Our system includes a vNSF that allows us to monitor the IoT traffic and, if needed, autonomously react when an anomaly is found. This component can perform two types of anomaly detection:

- *network attack identification*, where we detect attacks targeting an IoT device;
- *oblivious authentication*, where we can spot an IoT device mismatch (e.g., a thermal sensor behaving as a webcam).

Indeed, identifying an attack as soon as possible can help to react to and mitigate the undesired behavior promptly. Some attacks are, however, more challenging to spot. Expert attackers may try to hide their attempts by passing off their malicious network traffic as benign traffic. However, thanks to the oblivious authentication mechanisms, we can detect if a network flow related to a particular device starts behaving as another one.

To design this component, we extracted various aggregated features for every traffic flow using NFStream^5^. We then feed these values to a machine-learning model for the flow classification. In our implementation, we used some random forest classifiers implemented with scikit-learn^6^. We experimentally proved that this approach achieves promising results (see Section 4). In particular, we trained two different random forests:

- a coarse-grained classifier that distinguishes various attack categories (e.g., brute force, DDoS, DoS, spoofing) and device categories (i.e., audio, camera, home automation, power outlet), for a total of 15 classes;
- a fine-grained classifier that pinpoints the exact attack (e.g., SYN flood, SQL injection) or device (e.g., Amazon Alexa Echo Dot, Wemo Smart Plug), totaling 80 classes.

Users can choose among the classifiers above through a configuration parameter when deploying this module.

To train and test all our classifiers, we used the freely available CIC IoT Dataset 2023^7^. The paper by Neto et al. [39] contains the complete list of attacks, devices, and IoT categories we used in our approach.

Our traffic analysis approach always performs a Shallow Packet Inspection (SPI) by only looking at the network (e.g., IP) and transport (e.g., TCP, UPD, and ICMP) level headers without analyzing the payloads. Thus, our solution complies with various privacy-sensitive scenarios. Furthermore, it is also encryption-agnostic, allowing our approach to work equally well with both encrypted (e.g., TLS or WS-Security) and clear connections.

Table 1 lists all the 59 flow features we used. Most features come in three flavors: client-to-server, server-to-client, and global (round-trip) versions. The only features that are global connection properties are related to the protocols; in fact, we distinguish between IPv4 and IPv6, but also between some transport-layer protocols such as TCP, UDP, and ICMP.

**Figure 2:**
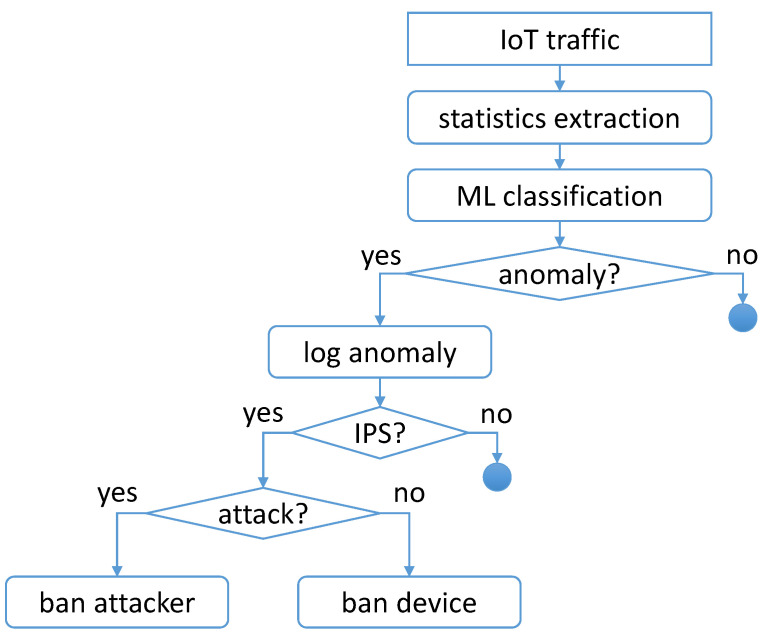
IDS/IPS module workflow.

This module can be configured as an IDS or an IPS, and its internal workflow is depicted in Figure 2. In the IDS variant, the module logs the anomalous flow in a file that can be used as a simple log or actively watched to perform a real-time analysis of the IoT devices. When configured as an IPS (in addition to the logging), this component can automatically add a DROP rule to the iptables firewall to block the IP address related to the anomalous connection. When an attack targeting an IoT device is detected, the IPS will block the external IP address (i.e., the attacker itself). On the other hand, in case of an oblivious authentication failure, the module will block the IoT device’s IP address. It is important to note that, due to how the Docker ecosystem works, the iptables kernel module that we are configuring is the host machine’s one since containers are without a proper kernel.

## 4. Experimental Results

In this section, we will discuss the results we obtained by experimentally testing our solution on various real traffic. In particular, Section 4.1 contains an investigation of the quality of our machine learning-based IDS/IPS vNSF, while Section 4.2 is a study of how much the network throughput is affected by the IoT Proxy.

### 4.1. Machine Learning Model Performance

As Section 3.2 explained, we trained two distinct random forests. In the following paragraphs, we will analyze the dataset we used, and then we will show some classification metrics showing the feasibility of our approach.

#### 4.1.1. Dataset

We used the PCAP files from the CIC IoT Dataset 2023 [39] from the University of New Brunswick to train and test our machine learning models. In our work, we recall that a sample is a network flow; this can be a traditional TCP connection [40], a QUIC connection [41], or just a simple ICMP packet [42].

We used the file names to identify the attacks in the dataset PCAP collection and the MAC addresses to split the benign capture files according to their devices, following the data published by Neto et al. [39]. Some PCAP files contained several benign flows with an unknown MAC address. We decided to group these flows in an ad hoc class called benign unknown to maximize the samples available for training and testing.

Our final dataset contains 129,092,256 samples (flows). Table 2 lists the number of flows by protocol. Most flows are IPv4 TCP connections belonging to DoS and DDoS attacks. UDP and ICMP flows are also present, mainly due to some UDP and ICMP attacks and QUIC connections.

Table 3 reports the sample counts for the attack and device categories used by our coarse-grained classifier. Due to their flooding nature, DoS and DDoS flows dominate the dataset. Benign connections are only 0.39% of the entire data set. This meager fraction will partially impact the detection accuracy of some device categories and types for the oblivious authentication mechanism, as described in the following paragraphs.

Table 4 shows instead some means computed on four different bidirectional features: flow duration, exchanged bytes, Package Inter-Arrival Time (PIAT), and the number of TCP SYN packets.

By performing a cursory analysis, several traffic patterns are already noticeable. For instance:

- lightning and power outlet connections are the longest ones since these devices are usually online for several minutes or hours;
- recon attacks send very few bytes per connection, and this behavior is consistent with how port and network scanning works;
- Mirai attacks [6] send vast quantities of bytes per connection since they are used to flood devices;
- DoS attacks send many TCP SYN packets since many of these attacks try to open as many channels as possible;
- DDoS attacks have a long PIAT since many of these attacks try to cause congestion by deliberately opening slow connections.

We used 80% of the dataset (103,273,804 samples) and the remaining 20% (25,818,452 samples) to train and test our models. We used a stratified split on the class attacks and devices to have more balanced train and test partitions.

#### 4.1.2. Coarse-Grained Classifier

We trained a random forest to distinguish between 15 classes: 8 are related to benign activities (audio, camera, home automation, hub, lighting, next-gen, power outlet, and unknown), while 7 are attacks (brute force, DDoS, DoS, Mirai, recon, spoofing, and web attacks).

**Figure 3:**
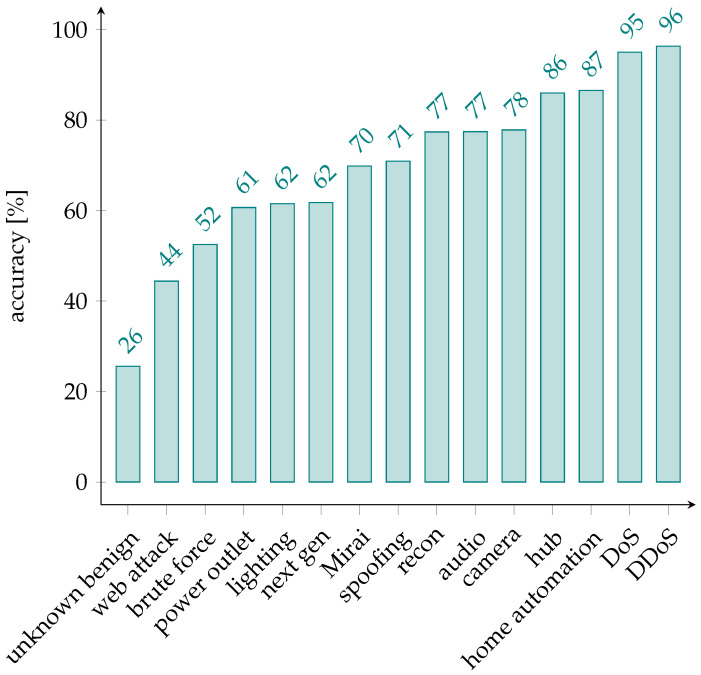
Class accuracies for the coarse-grained model.

Table 5 shows various classification metrics related to the coarse-grained classifier. Figure 3, instead, shows the accuracies per each class.

The balanced accuracy of the random forest is 69.56%, making our approach more than ten times more accurate than a random classifier^8^.

DDoS and DoS attacks are the easiest to spot for our classifier (with an accuracy of 96% and 95%, respectively). On the other hand, the unknown benign category is the hardest to detect; this might be caused by the variety of different flows belonging to this category, which might not be completely separable from the other ones.

#### 4.1.3. Fine-Grained Classifier

The fine-grained classifier is based on a random forest trained to distinguish among 80 classes, consisting of 33 attacks and 47 benign devices. Table 6 lists various classification statistics of this machine learning model.

The results obtained for this classifier are similar to the coarse-grained version, albeit slightly inferior. This decrease in accuracy is attributable to the fact that this is a significantly more challenging classification problem. Nonetheless, the balanced accuracy of our model is 60.38%, a significant achievement since the balanced accuracy of a random classifier is only $\frac{1}{80}=1.25\%$.

### 4.2. Overhead

We tested the network throughput degradation of the IoT proxy in our laboratory using the platform whose characteristics are listed in Table 7.

**Figure 4:**
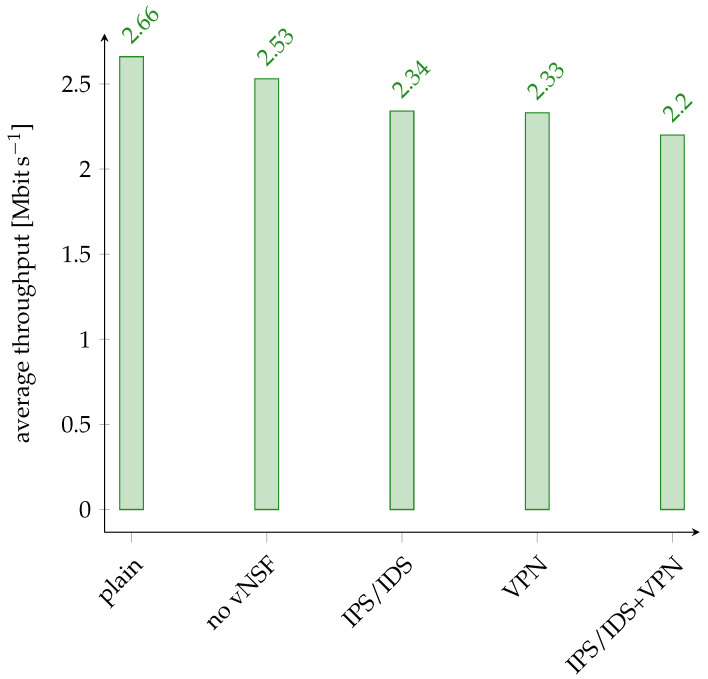
Overhead of the IoT proxy components.

We used iperf^9^ to estimate the bandwidth loss of the IoT proxy while communicating with a remote IoT device. Our results are reported in Figure 4, where we show respectively the bandwidth without the IoT proxy, with the IoT proxy without any active vNSF, by activating only the IPS/IDS vNSF, by activating only the VPN vNSF, and by enabling both.

In the IPS/IDS vNSF, most of the time is spent analyzing the packets with NFStream. We observed that using the coarse-grained or the fine-grained random forest does not affect performance degradation, so the data in Figure 4 are consistent with both approaches.

When the IoT proxy has all the vNSF enabled, the percentage loss of bandwidth is about 17%. Thanks to Docker’s lightweight virtualization technology, we can still maintain a good bitrate even with all IoT Proxy modules enabled, providing a good balance between security and performance for IoT devices.

## 5. Conclusions

As the number of connected Internet of Things (IoT) devices continues to rise, the inherent vulnerabilities in these devices have become a focal point for malicious activities, jeopardizing data integrity, user privacy, and even critical infrastructures. Thus, the security of IoT devices evolves from a technical necessity to a cornerstone for fostering innovation and trust in the digital age.

To provide security at the network’s edge, especially for resource-limited IoT devices, this paper introduces the *IoT Proxy*: a modular solution designed to fortify IoT devices’ security. The main goal of the IoT Proxy is to externalize security-related functions from the devices to a more powerful gateway equipped with Virtual Network Security Functions (VNSFs). This strategic offloading of security controls aims to mitigate the limitations of individual IoT devices, such as constrained computing power and memory.

The modular design of the IoT Proxy allows for the inclusion of various VNSFs. In the implemented proof of concept, we focused on two essential security functionalities: a Virtual Private Network (VPN) terminator and an Intrusion Prevention System (IPS). Notably, our IPS can identify the type of connected devices through a machine learning-based technique called *oblivious authentication*.

Experimental results from laboratory testing demonstrate promising outcomes, showcasing the efficacy of the IoT Proxy. The coarse-grained random forest achieves a balanced accuracy of around 70%, while the fine-grained model achieves a balanced accuracy of more than 60%. These results are respectively more than 10 times and about 50 times higher than that of a random classifier. Notably, it is interesting that the coarse-grained classifier can identify DoS and DDoS attacks with over 95% accuracy.

Thanks to its modular, scalable, and externalized security approach, the *IoT Proxy* can create a more resilient and secure IoT environment, especially for resource-limited IoT devices.

### Future Work

Several avenues for future research and development present themselves, expanding on the capabilities of the IoT Proxy and addressing emerging challenges in the dynamic landscape of IoT security.

One key direction for future work is the exploration of unsupervised machine learning models to enhance anomaly detection capabilities within the IoT Proxy. In particular, we intend to build upon the preliminary research we performed on the application of autoencoders for cyber-attack detection [43]. The system could adapt and identify novel threats by leveraging unsupervised learning techniques without relying on predefined attack datasets. This approach holds promise for detecting previously unseen attack patterns, contributing to the robustness of the intrusion detection mechanisms.

Live analysis of network traffic is another crucial feature we plan to research. In a previous research paper [44], we investigated the applications of several machine learning techniques, including random forests and neural networks, trained on a dataset we produced [45], to perform live detection of cyber attacks in multiple web scenarios. Adapting this approach to IoT scenarios and coupling it with the IoT Proxy could lead to an omni-comprehensive IoT security solution, able to adapt to evolving network behaviors and proactively guard against sophisticated and adaptive threats. Furthermore, adopting sequence models, such as Long Short-Term Memory (LSTM) networks and transformers, could enable a more accurate assessment of traffic patterns.

Moreover, the diversification of the datasets used for training and evaluation is paramount. Future research should involve the exploration of other IoT datasets, encompassing a wide range of devices and network scenarios. This approach ensures the generalizability and effectiveness of the IoT Proxy across diverse IoT ecosystems, each presenting unique challenges and security considerations.

Another aspect that we plan to consider is the validity of the choices taken by the IoT Proxy when multiple vNSFs are chained. Indeed, we have already investigated, in classical network security scenarios, approaches to formally model policies of both physical and virtualized network security appliances [46]. Such techniques could be specialized and integrated into the IoT Proxy to continuously ensure the coherence of the deployed vNSFs configurations with the high-level intents of IoT network administrators.

To adapt our solution to different IoT scenarios, such as 5G or 6G scenarios where devices are directly connected to the Internet, a further engineering approach should be considered. In these cases, a potential solution could be the deployment of a cloud-based IoT Proxy. The devices could be securely connected to the IoT Proxy through a VPN.

In conclusion, future work involves refining and expanding the existing functionalities of the IoT Proxy and delving into advanced techniques such as unsupervised learning, live traffic analysis, and diverse dataset utilization. These endeavors aim to fortify the IoT Proxy’s capabilities in the face of ever-changing IoT security challenges.

## Figures and Tables

**Table 1:** Features for the flow classification. The checkmark means that the specific feature is considered during the training phase.

Description	Unit	Global	Sent	Received
flow duration	ms	√	√	√
packet count	packet	√	√	√
byte count	byte	√	√	√
minimum packet size	byte	√	√	√
mean packet size	byte	√	√	√
st. deviation of packet size	byte	√	√	√
maximum packet size	byte	√	√	√
minimum inter-arrival time	ms	√	√	√
mean inter-arrival time	ms	√	√	√
st. deviation of inter-arrival time	ms	√	√	√
maximum inter-arrival time	ms	√	√	√
TCP packets with SYN set	packet	√	√	√
TCP packets with CWR set	packet	√	√	√
TCP packets with ECE set	packet	√	√	√
TCP packets with ACK set	packet	√	√	√
TCP packets with PSH set	packet	√	√	√
TCP packets with RST set	packet	√	√	√
TCP packets with FIN set	packet	√	√	√
use of IPv4	boolean	√	-	-
use of IPv6	boolean	√	-	-
use of TCP	boolean	√	-	-
use of UDP	boolean	√	-	-
use of ICMP	boolean	√	-	-

**Table 2:** Flow count by protocol.

Protocol	IPv4	IPv6
TCP	124,028,344	6
UDP	4,878,325	46,212
ICMP	89,958	0
other	14,065	35,330
total	129,010,692	81,564

**Table 3:** Flow counts by attack and device category.

(**a**) Benign traffic.	
**Category**	**Flows**
audio	48,717
camera	126,364
home automation	10,630
hub	20,252
lighting	1323
next-gen	173
power outlet	2140
unknown	297,238
total	507,002
(**b**) Malicious traffic.	
**Category**	**Flows**
brute force	6452
DDoS	118,780,042
DoS	7,978,026
Mirai	512,905
recon	1,141,540
spoofing	147,823
web attack	18,466
total	128,585,254

**Table 4:** Some mean statistics by attack and device category.

Category	Duration [ms]	Bytes	PIAT [ms]	SYN Packets
audio	79,819.59	7257.89	1481.24	0.55
brute force	45,737.65	5662.02	1689.59	0.93
camera	31,344.17	44,393.27	806.44	0.22
DDoS	318,543.99	2314.38	22,238.50	6.40
DoS	427,393.55	7751.54	8180.93	24.55
home automation	120,800.44	6186.60	2436.34	0.51
hub	67,226.78	3951.05	2446.75	0.41
lighting	942,282.45	20,572.59	12,207.54	0.37
Mirai	23,256.00	288,397.63	1785.00	0.93
next-gen	1878.77	79,213.42	232.80	0.04
power outlet	693,913.67	14,842.67	7144.68	0.28
recon	12,464.81	1447.62	499.59	0.89
spoofing	32,446.62	24,107.19	3053.01	0.34
unknown	50,424.88	22,804.24	1303.65	0.49
web attack	33,310.13	5133.73	3222.72	0.43

**Table 5:** Classification statistics for the coarse-grained model.

Statistic	Value [%]
accuracy	95.72817533754541
balanced accuracy	69.56422432885422
AUC	96.51575656890216
*F*-score (macro)	35.80059247737348
recall (macro)	69.56422432885422
precision (macro)	28.473269071998175

**Table 6:** Classification statistics for the fine-grained model.

Statistic	Value [%]
accuracy	96.0684655558229
balanced accuracy	60.3814098614751
AUC	94.34290472146797
*F*-score (macro)	33.337772924857084
recall (macro)	60.3814098614751
precision (macro)	28.746583759039684

**Table 7:** Features of our test platform.

Component	Version
operating system	GNU/Linux Ubuntu 20.04.1
kernel	5.15.0-72-generic
Python	3.8.10
Docker	20.10.21 (build baeda1f)
docker-compose	1.25.0
scikit-learn	1.1.0
CPU	Intel® Core™ i7-1065G7 CPU @ 1.30GHz
RAM	8 GiB

## Data Availability

To train the machine learning models, we used the IoT traffic dataset provided by the Canadian Institute for Cybersecurity (CIC). The CICIoT Dataset 2023 is publicly available at https://www.unb.ca/cic/datasets/iotdataset-2023.html (accessed on 7 October 2023).

## Footnotes

1 https://fishy-project.eu/, accessed on 2 October 2023.

2 https://www.eeas.europa.eu/eeas/horizon-2020_en, accessed on 2 October 2023.

3 https://www.strongswan.org/, accessed on 3 October 2023.

4 See note 3 above.

5 https://www.nfstream.org/, accessed on 6 October 2023.

6 https://scikit-learn.org/, accessed on 6 October 2023.

7 https://www.unb.ca/cic/datasets/iotdataset-2023.html, accessed on 7 October 2023.

8 The balanced accuracy of a 15-class classifier is $\frac{1}{15}∼6.66\%$.

9 https://iperf.fr/, accessed on 14 November 2023.

## Abbreviations

The following abbreviations are used in this manuscript:

AUC	Area Under the Curve
CIC	Canadian Institute for Cybersecurity
CIoT	Consumer Internet of Things
DDoS	Distributed Denial of Service
DoS	Denial of Service
DPI	Deep Packet Inspection
GA	Genetic Algorithm
HTTP	Hypertext Transfer Protocol
HTTPS	Hypertext Transfer Protocol Secure
ICMP	Internet Control Message Protocol
IDS	Intrusion Detection System
IIoT	Industrial Internet of Things
IKE	Internet Key Exchange
IoT	Internet of Things
IP	Internet Protocol
IPsec	Internet Protocol Security
IPS	Intrusion Prevention System
LSTM	Long Short-Term Memory
MAC	Media Access Control
NIDS	Network-based Intrusion Detection System
PDAE	Parallel Deep Auto-Encoder
PIAT	Package Inter-Arrival Time
PKI	Public Key Infrastructure
QUIC	Quick UDP Internet Connections
RF	Random Forest
SPI	Shallow Packet Inspection
TCP	Transmission Control Protocol
TLS	Transport Layer Security
UDP	User Datagram Protocol
vNSF	virtual Network Security Function
VPN	Virtual Private Network
